# The Book World of Medicine and Science

**Published:** 1906-07-14

**Authors:** 


### The Book World of Medicine and Science.

Manual of Anatomy, Systematic and Practical, including Embryology. By A. M. Buchanan, M.A., M.D., C.M., F.F.P.S.Glas., Professor of Anatomy in Anderson's College, Glasgow, Examiner in Anatomy for the Triple Qualification of the Scottish Licensing Bodies, for the Dental Diploma, and for the Fellowship of the Faculty of Physicians and Surgeons of Glasgow. Vol. I. Osteology, Upper Limb, Lower Limb. With 268 illustrations, mostly original and in colours. University Series. (London: Ballière, Tindall and Cox, 8 Henrietta Street, Covent Garden. 1906. All rights reserved. Pages 596. Price 12s. 6d.)

This important Manual of Anatomy is very properly dedicated to Lord Lister in grateful acknowledgment of the powerful influence which he exercised over the intellectual culture of his Glasgow students, of whom the author was one. Professor Buchanan has done credit to his early culture, and the first volume of the work is marked by a refinement of style, an exactitude of expression, a beautiful simplicity of arrangement, a fullness of material knowledge, and an unrivalled lucidity of language worthy of a great master. The Manual is quite devoid of the intricate embellishments with which modern editors of older works have succeeded in encumbering them. Their very size and price, not to speak of innumerable annotations and footnotes, have created in the student at the outset of his career a feeling of consternation, repugnance, and self-commiseration. To him, therefore, as well as to the pure anatomist, this work will be welcome. Professor Buchanan's object has been to combine in quite a new method a manual of practical anatomy with a systematic text-book, and to furnish students not with a new edition of some older author rendered further obscure and intricate by modern editors, but with a complete treatise on the subject composed and written entirely by himself. The author has accomplished his task in a scholarly and scientific fashion, and presents us with an anatomical treatise which will be treasured not only by the student as an easy highway to a place of honour on the examination list, but—and this is of greater importance—by the general practitioner of medicine and surgery who requires anatomical points elucidated with fullness, clearness, precision, and point, for the breathing-spaces he may have to devote to studious work. The section on Osteology is treated of in an exhaustive manner in the present volume. An account of the ossification of each bone follows its minute description. The section is of extreme interest to every practitioner—to the gynæcologist, the dentist, and the special practitioner. The drawings in this section amount in number to 257, and are beautifully executed, accurate in every detail of muscular attachments and other bone markings. The origins and insertions of muscles are delineated in different colours in these drawings, specially executed for this work, the origins being in red and the insertions in blue. From a study of this section alone it is easy to see that the author has not spared himself, but has put much labour into the volume. We feel his work deserves the warmest welcome, and, in fact, is certain to receive it.

The section on Arthrology is short, only grouping together the definitions and terms, leaving the description of the joints to come in their natural order—the order in which they are met in dissection or in operation. The descriptions then given are full, and are amply illustrated by a series of specially prepared drawings, which have been executed by the well-known anatomical artist, Mr. James T. Murray, of Edinburgh, who has done his work—as the author has done his—with the greatest care, accuracy, and ability. To the section dealing with the upper limb is appended a complete guide to its dissection, and this is a valuable and distinguishing characteristic of the work. The same thing happens in the case of the lower limb, abdomen, thorax, and head and neck. The dissecting guide gives full information as to the manner in which the dissections should be carried out, and details the structures which should be exposed. The author's able and lucid explanation of the enervation of the muscles and of their action is given very fully, not tabulated, and is thereby of the greatest use in view of the growing importance of a knowledge of the action of muscles in nervous diseases and in surgical work. The volume is so important that it would have been advantageously even more copiously illustrated. For instance, the trapezius, Latissimus dorsi, levator anguli scapulæ, and the rhomboids should have had a good illustration to themselves. The deep muscles of the back and the middle column of the erector spinæ and the trachelo mastoid are very clearly represented, so also are the multifidus spinæ and the levatores costarum muscles, and not unnecessarily complicated with colour printing.

In the lower limb and elsewhere the "landmarks" might have been illustrated. This would have made the work more interesting to art students, and probably the author may deal with this aspect of the subject in the second volume or in an addendum.

The author has made a particular point of dealing with the various bursæ and synovial sheaths, and some very excellent illustrations are given of these on pages 353, 369, 508, and 510. It is impossible to mark out all the interesting points in this new Manual of Anatomy. It is a new departure in anatomical science worthy of the great anatomical school which has produced anatomists like Liston, Syme, Fergusson, Barclay, Fyfe, Gordon, Robert Knox, Lonsdale, John Reid, Charles Bell, the Lizars, Goodsir, Struthers, Turner, Clelland, and our author, Alexander M. Buchanan. His pupils all over the world will welcome the book as a memento worthy in every way of a grand anatomist, an exquisite demonstrator, and a master of lucidity. We look forward with keen interest to the issue of the second volume of this Manual in September, when the author will deal with the anatomy of the abdomen, thorax, and head and neck, the minute anatomy of the viscera and organs, and with the nervous system, the eye, the ear, and the nose. The subject of embryology is to be dealt with in the same way as the author has dealt with the subject of ossification—namely, by appending to the description of each viscus a concise account of its development. In this way the attention of the student is sure to be attracted to this very important subject. We unhesitatingly recommend this work to the advanced student as a model of accuracy; to the practical physician and surgeon as a pattern of concise anatomical knowledge, to the beginner in medical studies as the easiest way of acquiring and the surest way of remembering anatomical detail, as well as to the artist and to the hospital nurse who takes an intelligent interest in the work of the operating-room. It may be added that throughout the whole work the author has, from his long experience not only as a teacher but as an examiner on various boards, kept constantly in view the examinational requirements of students, for whom the work is specially intended, and to whom it will prove in the highest degree the most helpful anatomical work hitherto published.

Preservatives in Food and Food Examination. By John C. Thresh, M.D., D.Sc., and Arthur E. Porter, M.D., M.A. (London: J. and A. Churchill. 1906. Pp. xv. + 456. Plates viii.)

The volume before us deals with the subject which at the present time is of great interest to all hygienists. It is divided into five parts, the first part deals exclusively with the physical, chemical, and pharmacological properties of chemical preservatives. Part II. deals with food and drinks themselves, their purity and the usual substances used to preserve them being fully considered. The foods dealt with under this head include milk, cream, butter, margarine, alcoholic beverages, temperance drinks, fruits, jams, and vegetables, meat, game, eggs and fish. Part III. is devoted to the question of colouring-matters in food and drink and to certain mineral poisons which may occur in articles of diet. Chief among these latter is arsenic, and Chapter XVI. includes an account of some of the chief epidemics of arsenical poisoning. Under this head, also, metallic contamination of canned foods is discussed, chiefly so far as concerns lead and tin. The question of copper in tinned green vegetables is also treated of.

Part IV. in its first chapter considers fully the question of the law relating to food inspection and the Public Health Acts of 1875 and 1890. Different varieties of unsound food are described in the ensuing chapters, and in Chapter XXV. an account is given of the food epidemics investigated by the Local Government Board. The immediate cause of these epidemic food-poisonings have only been investigated with any degree of completeness in comparatively recent years, and until lately the classification of the associated bacteria has been very imperfect. The author subsequently discusses the complicated question of ptomaine poisoning. The last part of the book deals fully with the detection and estimation of the chief chemical preservatives and the usual metallic impurities in food. Chapter XXIII. is devoted to the technique of food-examination for coal-tar colouring matters, and the subsequent two chapters to the appearances and deceptive characteristics of food and to the chief legal cases occurring under the Sale of Food and Drugs Act. Two appendices conclude the work, the former dealing with the Report of the Departmental Committee on the Use of Preservatives and Colouring Matters in Articles of Food and Drink, and the latter upon Law and Practice in certain Foreign Countries and the Colonies as to the Preservatives and Colouring Matters in Food.

Throughout the book constant reference is made to the Report of the Departmental Committee on Colouring Matter and Preservatives in Food, and it speaks volumes for the thoroughness and accuracy of the Committee's inquiries and original work that four years after its publication but little has been added to our knowledge in the subject-matter in question. The book will certainly be of value to all interested in the question of food adulteration, and although we have excellent books dealing fully with flesh foods and dairy produce from the same standpoint, we are not aware of any book in the English language covering so large an area of inquiry as the present volume. The standard work of Villiers and Collin in French deals certainly more exhaustively than the present one with a similar subject, and we think should have been mentioned in the preface, as also should the classical but now somewhat out-of-date treatise of Heimzerling on Die Conservirung der Nahrungs- und Genussmittel.

